# The combination of circulating levels of ANGPTL, omentin-1, leptin and cytokines is associated with polycystic ovary syndrome in different BMI groups

**DOI:** 10.61622/rbgo/2025rbgo86

**Published:** 2025-11-18

**Authors:** Rumeysa Çınar, Özlem Kayacık Günday, Ahmet Kahraman, Mehmet Yılmazer

**Affiliations:** 1 Afyonkarahisar University of Health Sciences Faculty of Medicine Department of Biochemistry Afyonkarahisar Turkey Afyonkarahisar University of Health Sciences, Faculty of Medicine, Department of Biochemistry, Afyonkarahisar, Turkey.; 2 Afyonkarahisar University of Health Sciences Faculty of Medicine Department of Obstetrics and Gynecology Afyonkarahisar Turkey Afyonkarahisar University of Health Sciences, Faculty of Medicine, Department of Obstetrics and Gynecology, Afyonkarahisar, Turkey.

**Keywords:** Polycystic ovary syndrome: Obesity, ANGPTL, Omentin-1, Leptin

## Abstract

**Objective:**

This study aims to explore the combined role of ANGPTL3, 4, 8, omentin-1, leptin, TNF-α, and IL-6 molecules known for their roles in fat metabolism, obesity, and inflammation, yet whose connection to PCOS is still debated in the development of PCOS.

**Methods:**

A prospective cross-sectional study involving PCOS patients (n=30) and BMI-matched controls (n=30) was conducted. Levels of ANGPTL3, 4, 8, omentin-1, leptin, TNF-α, and IL-6 were measured in peripheral venous blood samples.

**Results:**

When dividing both the PCOS and control groups into six BMI-based subgroups normal weight (20-24.9 kg/m^2^), slightly overweight (25-29.9 kg/m^2^), and obese (30-39.9 kg/m^2^) there were significant differences in levels of ANGPTL3 and 8, omentin-1, leptin, IL-6, and TNF-α (p<0.05). Comparison between the entire PCOS and control groups showed that CRP levels were significantly higher in the PCOS group (p<0.0001), while omentin-1 levels were significantly lower (p=0.022). Regression analysis, including ANGPTL3, 4, 8, IL-6, and TNF-α alongside CRP and omentin-1, indicated a significant model for PCOS (Nagelkerke R^2^=0.698, p<0.0001, PPV=80%, NPV=90%). In ROC analysis, the AUC for CRP and omentin-1 were significant (p<0.05; AUC=0.800-0.328).

**Conclusion:**

This study suggests a continuous interaction among ANGPTL, omentin-1, leptin, and cytokines in the etiopathogenesis of PCOS.

## Introduction

Polycystic ovary syndrome (PCOS) is an endocrine disorder affecting 5-20% of women of reproductive age, characterized by hyperandrogenism, ovulatory dysfunction with chronic oligo/anovulation, and polycystic ovarian morphology.^(1)^ Hyperandrogenism contributes to insulin resistance, which is present in both normal weight (NW) and obese (O) patients with PCOS. Androgens contribute to insulin resistance by increasing free fatty acid release from adipocytes, reducing glycogen synthesis in muscle tissue, and affecting body fat distribution.^(2)^ The ratio and distribution of body fat are crucial in the clinical presentation and severity of PCOS.^(3)^ Obesity, lipid layer thickening, hyperinsulinemia, and disrupted gonadotropin secretion lead to metabolic disturbances in PCOS patients, creating a vicious cycle of weight gain and disease progression.^(3)^ Approximately 44% of women with PCOS have central adiposity.^(4)^ and they often exhibit a state of low-grade chronic inflammation, marked by elevated levels of proinflammatory markers such as C-reactive protein CRP.^(5)^ However, in a meta-analysis, CRP was shown to be elevated in PCOS patients independent of obesity.^(6)^ Yilmaz et al**.**^(7)^ found no difference in CRP levels in obese and lean PCOS **patients** and proposed new markers for PCOS that are better predictors than CRP. Importantly, low-grade inflammation in PCOS patients plays an important role in the development of insulin resistance, type 2 diabetes mellitus and atherosclerosis.^(8)^

The angiopoietin-like proteins (ANGPTLs) family, structurally similar to angiopoietins, consists of eight members: ANGPTL1 through ANGPTL8. These proteins regulate plasma lipid levels by modulating lipoprotein lipase (LPL)-mediated hydrolysis of proteins, triglycerides (TG), and phospholipids.^(9)^ ANGPTL3, a key regulator of lipoprotein metabolism, is associated with dyslipidemia, insulin resistance, and cardiovascular disease risk.^(10)^ Similarly, ANGPTL4 plays a role in lipoprotein metabolism and angiogenesis.^(11)^ ANGPTL8, or betatrophin, increases TG levels and inhibits LPL activity in a dose-dependent manner when overexpressed in the liver.^(12)^ Given the significant impact of ANGPTLs on lipid metabolism, their association with various diseases has been studied. ANGPTL4, ANGPTL6, and ANGPTL8, in particular, are considered potential contributors to metabolic syndrome and PCOS.^(13–16)^ However there is very limited data in the literature on how these markers can be assessed (laboratory analysis) in PCOS patients and how the levels should be interpreted in each case. Güneş et al.^(13)^ found a significant increase in Angptl-4 levels and a significant decrease in omentin-1 levels in both obese and non-obese PCOS patients compared to healthy individuals. Calan et al.^(15)^ also reported that ANGPTL 8, also called betatrophin, was higher in PCOS patients compared to BMI-matched controls.^(15)^

Leptin, a proinflammatory adipokine, stimulates the secretion of tumor necrosis factor-α (TNF-α) and interleukin-6 (IL-6) from macrophages in adipose tissue, thereby promoting low-grade inflammation.^(17)^ High leptin levels are thought to be linked to oxidative stress and PCOS.^(18)^ Another adipokine, omentin, is produced by visceral stromal vascular cells and is encoded by two genes (1 and 2). The primary isoform in human plasma is omentin-1^(19)^ which is considered a "good" adipocytokine. Plasma omentin levels and omentin mRNA expression in visceral adipose tissue are reduced in obese individuals^(20)^ and omentin levels may serve as predictors of metabolic outcomes or comorbidities related to obesity.^(21)^ Since obesity is common in PCOS, alterations in adipokine levels and increased prevalence of metabolic syndrome are expected in PCOS patients.^(13)^ However, the complex pathogenesis of PCOS limits the use of markers related to fat metabolism and inflammation in diagnostic criteria, as a single serum marker may have low sensitivity and specificity in predicting PCOS. Therefore, molecules such as ANGPTL, which may have a possible contribution to PCOS but are currently not cost-effective, continue to be investigated. Based on these insights, we aimed to assess the impact of combined lipid and inflammation markers including ANGPTL3, 4, 8, omentin-1, leptin, TNF-α, IL-6, HOMA-IR and CRP on PCOS development across three different BMI groups.

## Methods

This cross-sectional study included 30 women aged 19-44 diagnosed with PCOS according to the Rotterdam 2003 diagnostic criteria and 30 healthy women matched by BMI. Data collection occurred between November 2021 and March 2023. The study was approved by the University of Health Sciences Clinical Research Medical Ethics Committee (approval number: 2021/11;476) and adhered to the principles of the Declaration of Helsinki. All participants provided written informed consent.

Exclusion criteria included pregnancy, use of hormonal or oral contraceptives for abnormal menstrual bleeding, malignancy, breastfeeding, type 1 or type 2 diabetes, Cushing's Syndrome, classical or non-classical congenital adrenal hyperplasia, adrenal gland tumors, other causes of hyperandrogenism such as androgen-producing ovarian tumors or virilization, thyroid disorders, active or chronic liver or kidney disease, acute infection, recent use of corticosteroids, immunosuppressants, anti-hyperlipidemic, anti-hypertensive, or anti-hyperglycemic medications within the last two months, and refusal to participate. The control group comprised 30 volunteer women aged 19-44 years, without any health issues, hirsutism, acne, or hyperandrogenism symptoms, with regular menstrual cycles (25-34 days, lasting 2-7 days), and with BMI compatible with the PCOS group. Patients over the age of 44 were excluded. Thus, postmenopausal patients with particularly confusing aspects were not included in the study.

Participants were classified according to BMI categories established by the World Health Organization: underweight; BMI ≤20 kg/m^2^, normal weight (NW); BMI 20-24.9 kg/m^2^, slightly overweight (SO); BMI 25-29.9 kg/m^2^, obese (O); BMI 30-39.9 kg/m^2^, and morbidly obese; BMI ≥40 kg/m^2^. Venous blood samples were collected from all participants in the early follicular phase (days 2-5 of spontaneous or progesterone-induced menstruation) after a 10-hour fast. On the sampling day, fasting glucose, HOMA-IR, CRP, HbA1C, ALT, AST, GGT, total cholesterol, LDL, HDL, and triglycerides were measured using the Roche Cobas 6000 autoanalyzer (Roche-Hitachi Diagnostics, Japan). Serum samples for ANGPTL3, 4, 8, leptin, omentin-1, TNF-α, and IL-6 were stored in Eppendorf tubes at −80°C until analysis. Once thawed, the samples were brought to room temperature and analyzed using ELISA kits (BT LAB Human ANGPTL, leptin, TNFα, omentin, IL-6 Elisa Kit; China) on the RT-2100C and RT-3100 analyzers (Rayto, China).

At the initial assessment, following a 10-hour fast, volunteers’ height (m), weight (kg), body fat (kg), muscle mass (kg), and water content (kg) were measured and calculated using the TANITA BC-601 device (Body Composition Analyzer (Tanita, Tokyo, Japan).^(22)^ The TANITA method is faster, more practical and more cost-effective. It also has a high patient acceptance rate because it does not use X-rays as in DXA.

BMI was determined in kg/m^2^. Waist circumference was measured at the level of the umbilicus, and hip circumference was measured at the level of the greater trochanter. The waist-to-hip ratio (WHR) was then calculated.

Insulin resistance was assessed using both the HOMA-IR and the fasting plasma glucose-to-insulin ratio. HOMA-IR: This was calculated using the formula: [Fasting Plasma Insulin (mIU/mL) × Fasting Plasma Glucose (mg/dL)/405]. A value above 2.7 indicated insulin resistance.^(23)^

Anthropometric measurements were recorded, and BMI was calculated by dividing weight by the square of height. Clinical hyperandrogenism was evaluated through hirsutism scoring, using the modified Ferriman-Gallwey System.^(24)^

The Shapiro-Wilk test was used to assess whether the data followed a normal distribution. Descriptive statistics were presented as mean ± standard deviation or median (minimum-maximum) for continuous variables and as counts (n) and percentages (%) for categorical variables. Data with a normal distribution were analyzed using the independent sample t-test, while data without a normal distribution were analyzed with the Mann-Whitney U test. For comparisons across multiple groups, the Kruskal-Wallis test was applied.

To examine the relationship between ANGPTL, omentin-1, leptin, CRP, cytokine levels, and PCOS development, Spearman correlation coefficients, and odds ratios (OR) were calculated using Spearman correlation analysis and multivariate logistic regression analysis. Multiple linear regression analyses were conducted to adjust for covariates and to identify independent associations between ANGPTL, omentin-1, leptin, CRP, cytokine levels, and age, BMI, HOMA-IR, insulin, HbA1C, and LDL. All independent variables in the multiple linear regression analysis were tested for multicollinearity, with a variance inflation factor (VIF)> 3 indicating multicollinearity. Due to detected multicollinearity, HOMA-IR and insulin were excluded from the regression model. A receiver operating characteristic (ROC) analysis was used to evaluate the diagnostic performance of PCOS variables, and the area under the ROC curve (AUC) was calculated. Statistical analyses were performed using the Statistical Program for Social Sciences (SPSS), version 22.0 (Chicago, IL, USA), with a significance level of p ≤ 0.05 for all tests.

## Results

**Figure 1:**
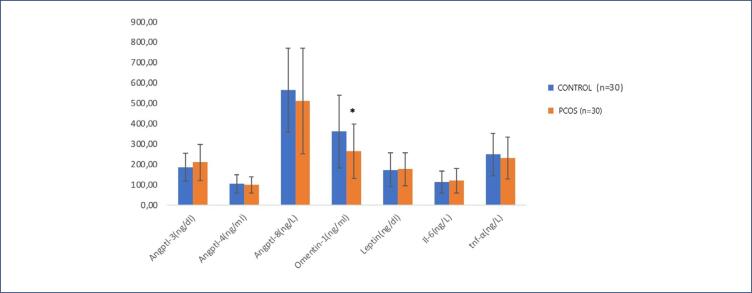
PCOS and control group ANGPTL, omentin, leptin and cytokine levels (*: p<0.05 compared to control group)

The study included 60 participants, comprising 30 women with PCOS (patient group) and 30 healthy women (control group). When comparing the PCOS and control groups, the PCOS group had significantly higher WHR and CRP levels (p=0.02 and p<0.001, respectively) and significantly lower omentin-1 levels (p=0.022) (Table 1) (Figure 1).

**Table 1:** Baseline characteristics, anthropometric measurements, and metabolic analysis for control (NW, SO, O) and PCOS (NW, SO, O) groups

Parameters	Control (n=30)				PCOS (n=30)				df	*p-level	**p-level
	All (n=30)	NW (n=10)	SO (n=10)	O (n=10)	All (n=30)	NW (n=10)	SO (n=10)	O (n=10)			
Age (years)	29.17 ± 8.91	23.7 ± 5.25	31.6 ± 8.25	32.2 ± 10.5	25.5 ± 4.53	23.5 ± 4.38	28.7 ± 4	24.3 ± 3.68	5	*0.018	0.235
BMI (kg/cm2)	27.09 ± 5.15	21.22 ± 2.11	27.36 ± 1.52	32.68 ± 2.39	28.08 ± 4.86	22.94 ± 1.56	27.64 ± 1.47	33.67 ± 2.71	5	*0.000	0.445
WHR	0.81 ± 0.08	0.75 ± 0.04	0.8 ± 0.06	0.88 ± 0.07	0.87 ± 0.07	0.83 ± 0.07	0.87 ± 0.06	0.92 ± 0.05	5	*0.000	**0.02
Body fat (kg)	25.19 ± 9.28	15.14 ± 5.28	26.73 ± 4.64	33.7 ± 5.75	26.74 ± 8.86	18.5 ± 4.61	25.5 ± 3.91	36.2 ± 6.32	5	*0.000	0.512
Body skelatal muscle (kg)	43.74 ± 5.34	38.6 ± 3.2	43.93 ± 3.91	48.7 ± 3.09	43.94 ± 4.67	39.16 ± 1.99	44.02 ± 2.81	48.64 ± 2.93	5	*0.000	0.880
HOMA-IR	2.79 ± 1.8	1.81 ± 0.59	1.69 ± 0.6	4.88 ± 1.59	3.28 ± 1.94	3.04 ± 1.22	2.32 ± 1.08	4.48 ± 2.59	5	*0.000	0.121
CRP (mg/L)	1.83 ± 2.01	0.38 ± 0.18	1.17 ± 0.59	3.95 ± 2.19	4.21 ± 3.59	3.44 ± 1.88	3.23 ± 1.84	5.96 ± 5.44	5	*0.000	0.000
Fasting insülin (mIU/mL)	12.27 ± 7.32	7.87 ± 2.29	8.11 ± 2.93	20.83 ± 6.06	14.04 ± 5.7	14.15 ± 5.74	10.51 ± 4.48	17.47 ± 4.98	5	*0.000	0.107
HbA1C %	5.43 ± 0.45	5.32 ± 0.29	5.29 ± 0.42	5.69 ± 0.53	5.43 ± 0.83	5.21 ± 0.28	5.13 ± 0.17	5.97 ± 1.28	5	*0.016	0.340
LDL (mg/dl)	108.3 ± 25.79	99.26 ± 17.08	107.7 ± 35.16	118.18 ± 20.37	107.71 ± 30.8	100.57 ± 36.19	94.55 ± 28.1	128.01 ± 15.81	5	*0.023	0.927
HDL (mg/dl)	51.05 ± 14.28	56.74 ± 11.04	58.24 ± 13.25	38.18 ± 9.03	49.53 ± 11.95	54.61 ± 10.18	43.36 ± 12.1	50.62 ± 11.75	5	*0.001	0.656
Triglycerides (mg/dl)	122.5 ± 77.47	73.07 ± 28.87	99.15 ± 59.08	195.55 ± 75.91	127.66 ± 45.86	107.93 ± 32.87	139.55 ± 47.45	135.49 ± 52.8	5	*0.000	0.234
Angptl-3 (ng/dl)	185.8 ± 67.59	224.55 ± 55.49	133.3 ± 52.46	199.78 ± 62.81	210.11 ± 88.24	179.77 ± 52.64	183.94 ± 47.17	266.62 ± 121.5	5	*0.017	0.442
Angptl-8 (ng/L)	565.5 ± 205.6	458.11 ± 106.46	675.6 ± 240.7	563.02 ± 201.47	511.29 ± 258.71	319.27 ± 128.14	456.59 ± 144.5	758.01 ± 257.75	5	*0.000	0.156
Omentin-1 (ng/ml)	361.9 ± 179.0	236.06 ± 89.15	463.0 ± 165.9	386.68 ± 195.42	265.11 ± 134.39	191.96 ± 48.29	186.71 ± 58.45	416.65 ± 118.98	5	*0.000	0.022
Leptin (ng/dl)	172.3 ± 83.73	102.25 ± 26.12	257.36 ± 56.8	157.52 ± 70.33	176.92 ± 80.85	168.52 ± 96.47	126.23 ± 40.01	236 ± 57.82	5	*0.000	0.728
IL-6 (ng/L)	112.99 ± 53.8	67.87 ± 10.38	167.29 ± 40.8	103.81 ± 43.82	119.43 ± 59.5	85.4 ± 32.75	81.71 ± 24.69	191.18 ± 33.74	5	*0.000	0.723
TNF-α (ng/L)	247.98± 103.0	148.46 ± 23.67	336.25 ± 35.3	259.22 ± 112.28	231.33 ± 102.23	159.99 ± 41.66	230.71 ± 115.23	303.3 ± 85.17	5	*0.000	0.595

All values are presented as mean (SD); Kruskal-Wallis Test for differences between control (NW, SO, O) and PCOS (NW, SO, O) groups (NW: normal weight, SO: slightly overweight, O: obese).

* Significant difference (p< 0.05): comparison of control (NW, SO, O) and PCOS (NW, SO, O) Groups; Student's t-test or Mann–Whitney U-Test for differences between control and PCOS groups

** Significant difference: comparison of control and PCOS Groups; PCOS: polycystic ovary syndrome; nw: normal weight; so: slightly overweight; o: obese; BMI: body mass index; WHR: waist-hip ratio; HOMA-IR: homeostasis model assessment insulin resistance; HDL: high-density lipoprotein; LDL: low-density lipoprotein; ANGPTL: Angiopoetin-like protein

No differences were observed in ANGPTL and cytokine parameters between these groups. However, when participants were further divided into six subgroups based on BMI normal weight (NW, 20-24.9 kg/m^2^), slightly overweight (SO, 25-29.9 kg/m^2^), and obese (O, 30-39.9 kg/m^2^) significant differences were found in all metabolic and biochemical parameters, including ANGPTL3, ANGPTL4, ANGPTL8, leptin, omentin-1, TNF-α, IL-6, CRP, LDL, HDL, HOMA-IR, WHR, body water, muscle, and fat ratio, across the groups (p<0.05) (Table 2).

**Table 2:** Comparison of parameters for control and PCOS patient groups

Parameters	Control		Obese		*p-value	**p-value
	All (n=30)	Obese control (n=10)	All (n=30)	O-PCOS (n=10)		
Angptl-3 (ng/dl)	185.8 ± 67.59	199.78 ± 62.81	210.11 ± 88.24	266.62 ± 121.5	*0.017	0.442
Angptl-8 (ng/L)	565.5 ± 205.6	563.02 ± 201.47	511.29 ± 258.71	758.01 ± 257.75	*0.000	0.156
Omentin-1 (ng/ml)	361.9 ± 179.0	386.68 ± 195.42	265.11 ± 134.39	416.65 ± 118.98	*0.000	0.022
Leptin (ng/dl)	172.3 ± 83.73	157.52 ± 70.33	176.92 ± 80.85	236 ± 57.82	*0.000	0.728
IL-6 (ng/L)	112.99 ± 53.8	103.81 ± 43.82	119.43 ± 59.5	191.18 ± 33.74	*0.000	0.723
TNF-α (ng/L)	247.98± 103.0	259.22 ± 112.28	231.33 ± 102.23	303.3 ± 85.17	*0.000	0.595

All values are presented as mean (SD); Kruskal-Wallis Test for differences between control (NW, SO, O) and PCOS (NW, SO, O) groups (NW: normal weight, SO: slightly overweight, O: obese).

* Significant difference (p< 0.05): comparison of control (NW, SO, O) and PCOS (NW, SO, O) Groups; Student's t-test or Mann–Whitney U-Test for differences between control and PCOS groups;

** Significant difference: p<0.05, comparison of control and PCOS groups

For the NW group, the PCOS subgroup showed significantly higher WHR, HOMA-IR, CRP, insulin, ALT, AST, and TG levels compared to the NW control group (p=0.009, 0.023, <0.001, 0.010, 0.030, 0.025, and 0.049, respectively). Additionally, ANGPTL8 levels were significantly lower in the NW PCOS group than in the NW control group (NW control: 458.11 ± 106.46, NW PCOS: 319.27 ± 128.14, p=0.008) (Table 3).

**Table 3:** Comparison of parameters for normal weight healthy woman and normal weight women with PCOS groups

Parameters	NW control (n=10)	NW PCOS (n=10)	p-level
Age (years)	23.7 ± 5.25	23.5 ± 4.38	0.849
BMI (kg/cm2)	21.22 ± 2.11	22.94 ± 1.56	0.05
WHR	0.75 ± 0.04	0.83 ± 0.07	*0.009
Body fat (kg)	15.14 ± 5.28	18.5 ± 4.61	0.199
Body water (kg)	29.99 ± 2.6	30.52 ± 1.62	0.290
Body skelatal muscle (kg)	38.6 ± 3.2	39.16 ± 1.99	0.344
Fasting plasma glucose (mg/dl)	92.11 ± 5.77	87.13 ± 5.72	0.054
HOMA-IR	1.81 ± 0.59	3.04 ± 1.22	*0.023
CRP (mg/L)	0.38 ± 0.18	3.44 ± 1.88	*0.000
Fasting insülin (mIU/mL)	7.87 ± 2.29	14.15 ± 5.74	*0.010
HbA1C %	5.32 ± 0.29	5.21 ± 0.28	0.449
ALT (U/L)	10.8 ± 4.26	17 ± 8.88	*0.030
AST (U/L)	14.6 ± 4.01	20.9 ± 8.27	*0.025
GGT (U/L)	10.3 ± 3.59	12.5 ± 5.54	0.426
Total cholesterol (mg/dl)	158.11 ± 21.7	166.59 ± 39.99	0.290
LDL (mg/dl)	99.26 ± 17.08	100.57 ± 36.19	0.940
HDL (mg/dl)	56.74 ± 11.04	54.61 ± 10.18	0.344
Triglycerides (mg/dl)	73.07 ± 28.87	107.93 ± 32.87	*0.049
Angptl-3 (ng/dl)	224.55 ± 55.49	179.77 ± 52.64	0.082
Angptl-4 (ng/ml)	87.06 ± 19.95	81.74 ± 21.66	0.406
Angptl-8 (ng/L)	458.11 ± 106.46	319.27 ± 128.14	*0.008
Omentin-1 (ng/ml)	236.06 ± 89.15	191.96 ± 48.29	0.257
Leptin (ng/dl)	102.25 ± 26.12	168.52 ± 96.47	0.112
IL-6 (ng/L)	67.87 ± 10.38	85.4 ± 32.75	0.406
TNF-α (ng/L)	148.46 ± 23.67	159.99 ± 41.66	0.545

All values are presented as mean (SD); Student's t-test or Mann–Whitney U-Test for differences between nw control and nw PCOS groups

* Significant difference: p<0.05

In the SO group, PCOS participants had significantly higher WHR, CRP, and TG levels, and lower HDL levels compared to SO controls (p=0.023, 0.001, 0.041, and 0.034, respectively). The SO PCOS group also had significantly higher ANGPTL3 levels (PCOS: 183.94 ± 47.17, control: 133.35 ± 52.46, p=0.049) but lower levels of ANGPTL8, omentin-1, leptin, IL-6, and TNF-α (p=0.041, <0.001, <0.001, <0.001, and 0.023, respectively) (Table 4).

**Table 4:** Comparison of parameters for slightly overweight control and slightly overweight PCOS groups

Parameters	SO control (n=10)	SO PCOS (n=10)	p-level
Age (years)	31.6 ± 8.25	28.7 ± 4	0.343
BMI (kg/cm2)	27.36 ± 1.52	27.64 ± 1.47	0.496
WHR	0.8 ± 0.06	0.87 ± 0.06	*0.023
Body fat (kg)	26.73 ± 4.64	25.5 ± 3.91	0.364
Body water (kg)	34.4 ± 3.31	34.51 ± 2.31	0.650
Body skelatal muscle (kg)	43.93 ± 3.91	44.02 ± 2.81	0.650
Fasting plasma glucose (mg/dl)	85.14 ± 5.95	87.3 ± 6.46	0.307
HOMA-IR	1.69 ± 0.6	2.32 ± 1.08	0.199
CRP (mg/L)	1.17 ± 0.59	3.23 ± 1.84	*0.001
Fasting insülin (mIU/mL)	8.11 ± 2.93	10.51 ± 4.48	0.257
HbA1C %	5.29 ± 0.42	5.13 ± 0.17	0.384
ALT (U/L)	14 ± 8.19	17.4 ± 7.07	0.306
AST (U/L)	15 ± 4.76	18.6 ± 4.3	0.078
GGT (U/L)	8.7 ± 2.16	18.2 ± 11.6	*0.006
Total cholesterol (mg/dl)	173.4 ± 41.51	157.53 ± 32.19	0.326
LDL (mg/dl)	107.71 ± 35.16	94.55 ± 28.1	0.650
HDL (mg/dl)	58.24 ± 13.25	43.36 ± 12.1	*0.034
Triglycerides (mg/dl)	99.15 ± 59.08	139.55 ± 47.45	*0.041
Angptl-3 (ng/dl)	133.35 ± 52.46	183.94 ± 47.17	*0.049
Angptl-4 (ng/ml)	108.86 ± 44.86	83.44 ± 24.04	0.112
Angptl-8 (ng/L)	675.64 ± 240.79	456.59 ± 144.5	*0.041
Omentin-1 (ng/ml)	463.05 ± 165.97	186.71 ± 58.45	*0.000
Leptin (ng/dl)	257.36 ± 56.8	126.23 ± 40.01	*0.000
IL-6 (ng/L)	167.29 ± 40.86	81.71 ± 24.69	*0.000
TNF-α (ng/L)	336.25 ± 35.3	230.71 ± 115.23	*0.023

All values are presented as mean (SD); Student's t-test or Mann–Whitney U-Test for differences between control and PCOS groups

* (Significant difference)

No significant correlations were found between ANGPTL3 and ANGPTL8 and HOMA-IR, insulin, fasting glucose, LDL, HDL, TG, and total cholesterol (p>0.05). However, significant positive correlations were observed between ANGPTL4 and HOMA-IR and insulin, between omentin-1 and CRP, HbA1C, LDL, TNF-α, IL-6, and total cholesterol, and between leptin and HbA1C, LDL, and total cholesterol (p<0.05). Leptin and ANGPTL4 correlated positively with BMI, and omentin-1 and ANGPTL8 correlated positively with BMI, WHR, body fat-muscle, and water ratio (p<0.05). In the obese PCOS group (BMI >30 kg/m^2^), AST, HDL, leptin, and IL-6 levels were significantly higher compared to the obese control group (p=0.07, 0.013, 0.041, and <0.001, respectively) (Table 5).

**Table 5:** Comparison of parameters for obese control and obese PCOS groups

Parameters	O control) (n=10)	O PCOS (n=10)	p-level
Age (years)	32.2 ± 10.5	24.3 ± 3.68	0.138
BMI (kg/cm2)	32.68 ± 2.39	33.67 ± 2.71	0.496
WHR	0.88 ± 0.07	0.92 ± 0.05	0.212
Body fat (kg)	33.7 ± 5.75	36.2 ± 6.32	0.326
Body water (kg)	38.43 ± 2.6	38.6 ± 2.52	0.821
Body skelatal muscle (kg)	48.7 ± 3.09	48.64 ± 2.93	0.880
Fasting plasma glucose (mg/dl)	94.1 ± 7.35	101.06 ± 31.11	0.449
HOMA-IR	4.88 ± 1.59	4.48 ± 2.59	0.226
CRP (mg/L)	3.95 ± 2.19	5.96 ± 5.44	0.545
Fasting insülin (mIU/mL)	20.83 ± 6.06	17.47 ± 4.98	0.290
HbA1C %	5.69 ± 0.53	5.97 ± 1.28	1.0
ALT (U/L)	16 ± 5.21	25.4 ± 16.13	0.111
AST (U/L)	14.8 ± 2.04	25.2 ± 14.49	*0.007
GGT (U/L)	21.4 ± 7.56	20.4 ± 13.81	0.363
Total cholesterol (mg/dl)	173.89 ± 30.47	196.83 ± 21.48	0.096
LDL (mg/dl)	118.18 ± 20.37	128.01 ± 15.81	0.496
HDL (mg/dl)	38.18 ± 9.03	50.62 ± 11.75	*0.013
Triglycerides (mg/dl)	195.55 ± 75.91	135.49 ± 52.8	0.059
Angptl-3 (ng/dl)	199.78 ± 62.81	266.62 ± 121.5	0.131
Angptl-4 (ng/ml)	119.39 ± 59.28	132.41 ± 48.05	0.496
Angptl-8 (ng/L)	563.02 ± 201.47	758.01 ± 257.75	0.082
Omentin-1 (ng/ml)	386.68 ± 195.42	416.65 ± 118.98	0.496
Leptin (ng/dl)	157.52 ± 70.33	236 ± 57.82	*0.041
IL-6 (ng/L)	103.81 ± 43.82	191.18 ± 33.74	*0.000
TNF-α (ng/L)	259.22 ± 112.28	303.3 ± 85.17	0.406

All values are presented as mean (SD); Student's t-test or Mann–Whitney U-Test for differences between control and PCOS groups

* (Significant difference)

Comparing obese (O) and non-obese (NO) PCOS groups, the obese group had significantly higher levels of BMI, WHR, body fat, body water, body muscle, HOMA-IR, insulin, HbA1C, total cholesterol, LDL, ANGPTL4, ANGPTL8, omentin-1, leptin, IL-6, and TNF-α (p<0.001, 0.005, <0.001, <0.001, <0.001, 0.020, 0.018, 0.006, 0.007, 0.002, 0.006, 0.001, <0.001, 0.006, <0.001, and 0.006, respectively) (Table 6).

**Table 6:** Comparison of parameters for nonobese PCOS and obese PCOS groups

Parameters	NO PCOS (n=10)	O PCOS (n=10)	p-level
Age (years)	26.1 ± 4.88	24.3 ± 3.68	0.320
BMI (kg/cm2)	25.29 ± 2.82	33.67 ± 2.71	*0.000
WHR	0.85 ± 0.07	0.92 ± 0.05	*0.005
Body fat (kg)	22 ± 5.5	36.2 ± 6.32	*0.000
Body water (kg)	32.51 ± 2.82	38.6 ± 2.52	*0.000
Body skelatal muscle (kg)	41.59 ± 3.44	48.64 ± 2.93	*0.000
Fasting plasma glucose (mg/dl)	87.22 ± 5.94	101.06 ± 31.11	0.612
HOMA-IR	2.68 ± 1.18	4.48 ± 2.59	*0.020
CRP (mg/L)	3.33 ± 1.81	5.96 ± 5.44	0.179
Fasting insülin (mIU/mL)	12.33 ± 5.35	17.47 ± 4.98	*0.018
HbA1C %	5.17 ± 0.23	5.97 ± 1.28	*0.006
ALT (U/L)	17.2 ± 7.82	25.4 ± 16.13	0.158
AST (U/L)	19.75 ± 6.52	25.2 ± 14.49	0.426
GGT (U/L)	15.35 ± 9.32	20.4 ± 13.81	0.140
Total cholesterol (mg/dl)	162.06 ± 35.64	196.83 ± 21.48	*0.007
LDL (mg/dl)	97.56 ± 31.69	128.01 ± 15.81	*0.002
HDL (mg/dl)	48.99 ± 12.32	50.62 ± 11.75	0.912
Triglycerides (mg/dl)	123.74 ± 42.91	135.49 ± 52.8	0.523
Angptl-3 (ng/dl)	181.86 ± 48.7	266.62 ± 121.5	0.173
Angptl-4 (ng/ml)	82.59 ± 22.28	132.41 ± 48.05	*0.006
Angptl-8 (ng/L)	387.93 ± 150.44	758.01 ± 257.75	*0.001
Omentin-1 (ng/ml)	189.34 ± 52.25	416.65 ± 118.98	*0.000
Leptin (ng/dl)	147.37 ± 75.08	236 ± 57.82	*0.006
IL-6 (ng/L)	83.56 ± 28.29	191.18 ± 33.74	*0.000
TNF-α (ng/L)	195.35 ± 91.8	303.3 ± 85.17	*0.006

All values are presented as mean (SD); Student's t-test or Mann–Whitney U-Test for differences between control and PCOS groups

* (Significant difference)

**Figure 2:**
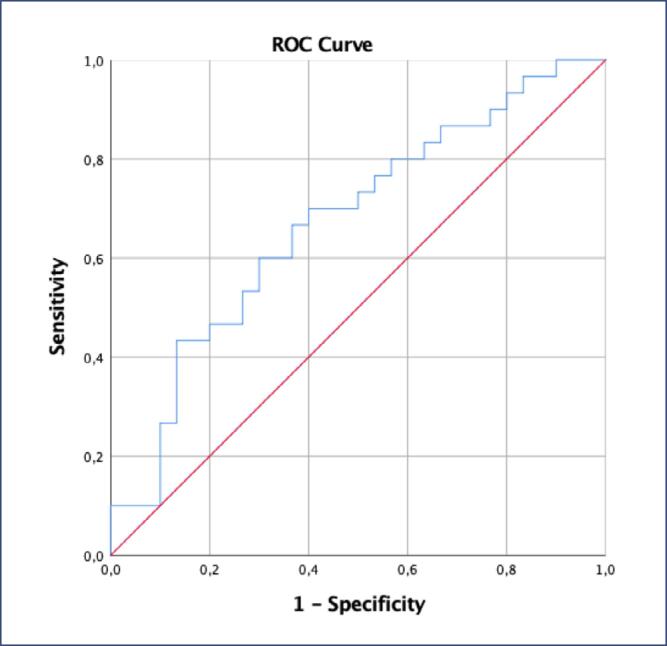
ROC (Receiver Operating Characteristic) analyses of omentin-1 for prediction of PCOS (AUC= 0.672, P= 0.022)

**Figure 3:**
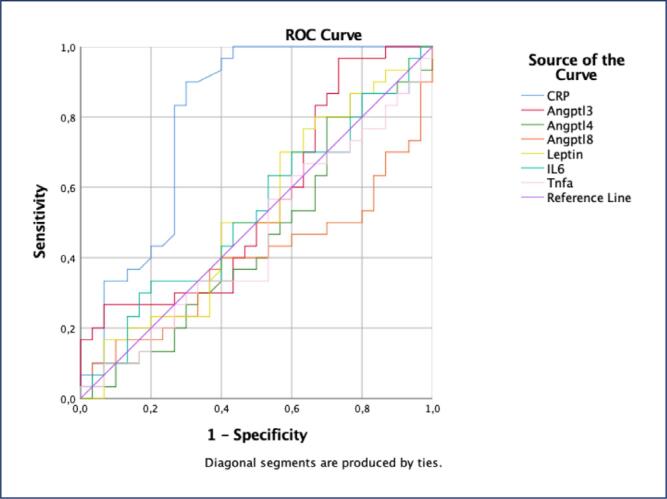
ROC (Receiver Operating Characteristic) analyses of ANGPTL 3, 4, 8, leptin, CRP and cytokine for prediction of PCOS (CRP: AUC= 0.800, P<0.0001)

Multiple linear regression analysis for covariates, including BMI, age, HOMA-IR, CRP, insulin, and HDL, was conducted before logistic regression to assess the impact of combined variables in predicting PCOS diagnosis. Due to multicollinearity, HOMA-IR and insulin were removed from the model. The model, which included CRP, LDL, ANGPTL3, 4, 8, omentin-1, leptin, IL-6, TNF-α, and BMI, was found to be significant in identifying patients at risk of developing PCOS (Nagelkerke R^2^: 0.698, omnibus tests of model coefficients <0.0001, PPV: 80%, NPV: 90%) (Table 7). The model showed that omentin-1 was significantly lower in PCOS patients, while leptin and CRP were significantly higher (p=0.015, 0.033, and 0.018, respectively) (Table 7). ROC analysis indicated significant AUC values for omentin-1 and CRP (AUC=0.672 and 0.800, respectively) (Figures 2 and 3).

**Table 7:** The odds ratio (OR) of PCOS status according to ANGPTL, Omentin1, Leptin, IL6, TNF-α, BMI, CRP, age and LDL levels using logistic regression analysis. Nagelkerke R Square: 0.698, Omnibus tests of model coefficients< 0.0001, PPV: 80 %, NPV: 90 %

Variable	p-value	β	OR	95% C.I.
CRP (mg/L)	0.018*	1.044	2.840	1.195- 6.747
LDL (mg/dl)	0.268	-0.025	0.976	0.934-1.019
Angptl-3 (ng/dl)	0.689	0.003	1.003	0.988- 1.019
Angptl-4 (ng/ml)	0.789	-0.003	0.997	0.972-1.022
Angptl-8 (ng/L)	0.608	-0.002	0.998	0.993-1.004
Omentin-1 (ng/ml)	0.015*	-0.035	0.966	0.939-0.993
Leptin (ng/dl)	0.033*	0.043	1.043	1.004-1.085
IL-6 (ng/L)	0.357	0.020	1.020	0.978- 1.064
TNF-α (ng/L)	0.930	-0.001	0.999	0.988- 1.011
BMI (kg/cm2)	0.817	-0.034	0.966	0.723- 1.292
Age (years)	0.180	-0.112	0.894	0.758-1053
Insulin resistance (1)	0.079	2.659	0.070	0.004- 1.359

* p-value of<0.05 was considered significant.

CI, confidence interval, PPV: Positive predictive value, NPV: Negative predictive value

## Discussion

Our study indicates that patients at risk of developing PCOS can be metabolically identified through a combination of ANGPTL3, 4, 8, omentin-1, leptin, IL-6, TNF-α, CRP, LDL, and BMI. Comparing the NW and SO PCOS patients with BMI-matched controls, we found that ANGPTL3 levels were higher in the PCOS group, while ANGPTL8 levels were lower (p<0.05). Omentin-1 levels were reduced across the entire PCOS group compared to controls and emerged as the most predictive parameter for PCOS (p<0.05).

Reviewing the literature, most studies have focused on single molecules, with ANGPTL8 being the most frequently studied among the ANGPTL proteins. There are also knowledge gaps regarding the roles of ANGPTL molecules in PCOS pathogenesis. The pathogenesis of PCOS, however, is highly complex, as are the impacts of metabolic pathways. Due to the long-term morbidity and metabolic syndrome caused by PCOS, early diagnosis and identification of new molecules that are cost-effective and contribute to prognosis are important, especially in developing countries.

Varikasuvu et al.^(25)^ recently reported higher circulating ANGPTL8 levels in PCOS patients compared to controls, regardless of BMI, and suggested that PCOS patients with greater insulin resistance had higher betatrophin concentrations. Other studies also reported elevated serum ANGPTL8 levels in PCOS.^(15,26–29)^

Previous research has suggested that ANGPTL8 plays a role in beta cell replication in insulin-resistant mouse models, though more recent studies have questioned these findings.^(30,31)^ One study proposed that elevated betatrophin levels in NO PCOS patients might represent a compensatory response to metabolic disturbances, though it remains unclear whether high betatrophin levels are a cause or consequence of PCOS.^(28)^ Contrary to these findings, Erbag et al.^(29)^ found that betatrophin levels were lower in PCOS patients compared to controls and that these levels were negatively correlated with BMI. Another study observed reduced betatrophin levels in patients with full-blown PCOS, particularly in those with a poor metabolic profile.^(32)^ Unlike these studies, which often investigated a single BMI group or did not include BMI-matched controls, our study found that ANGPTL8 levels were significantly lower in both NW and SO PCOS groups compared to their NW and SO control counterparts. When comparing all PCOS patients to controls without distinguishing by BMI, betatrophin levels were lower in the PCOS group, though not significantly (PCOS: 511.29 ± 258.71, control: 565.5 ± 205.6; p=0.156). ANGPTL8 levels were significantly higher in the obese PCOS (O PCOS) group compared to the non-obese PCOS (NO PCOS) group (O PCOS: 758.01 ± 257.75 vs. NO PCOS: 387.93 ± 150.44, p=0.001) and also higher in O PCOS compared to the obese control group, though not significantly (758.01 ± 257.75 vs. 563.02 ± 201.47; p>0.05). Consistent with our findings, Wu et al.^(33)^ reported higher serum ANGPTL8 levels in obese PCOS patients. Calan et al.^(15)^ also observed higher betatrophin levels in obese women compared to lean individuals, both in control and PCOS groups. Another study similarly reported significantly higher betatrophin levels in obese individuals, regardless of PCOS status, indicating a significant influence of BMI on serum betatrophin levels.^(34)^ Our findings support the notion that ANGPTL levels differ across BMI groups and have complex roles.

The literature also shows diverse correlations; for example, Calan et al.^(15)^ reported a positive correlation between betatrophin and HOMA-IR as well as free testosterone in PCOS patients, while Song et al.^(26)^ and Erbag et al.^(29)^ reported a negative correlation between serum betatrophin and BMI, fasting insulin, and HOMA-IR. In our study, we found no correlation between betatrophin and lipid or glucose profiles, but a significant positive correlation between ANGPTL8 and BMI, WHR, and body fat-muscle and water ratio.

Most research on ANGPTL3 has focused on conditions like type 2 diabetes and coronary artery disease.^(35,36)^ It has been reported that ANGPTL3, 4, and 8 are elevated in obesity and type 2 diabetes^(36)^ and that pharmacological inhibition of ANGPTL3 can reduce atherosclerotic lesion size.^(35)^ Cinkajzlová et al.^(37)^ found BMI to be an independent predictor of ANGPTL3 levels. Recent studies have shown significantly higher ANGPTL3 levels in women with PCOS compared to NO controls.^(16)^ In our study, ANGPTL3 levels were significantly higher in NW and SO PCOS patients than in controls. Circulating ANGPTL3 levels in PCOS patients were positively correlated with fasting glucose, fasting insulin, HOMA-IR, and triglycerides (TG), while showing a negative correlation with HDL, suggesting a potential direct link to dyslipidemia in PCOS patients.^(16)^ While no significant correlations were found between ANGPTL3 and glucose or lipid parameters, levels were generally higher in the PCOS group compared to controls (210.11 ± 88.24 vs. 185.89 ± 67.59; p>0.05). ANGPTL3 was also higher in the SO PCOS group than in the SO control group, aligning with the literature (p=0.049). In obese participants, ANGPTL3 levels were higher, though not significantly, in both the O PCOS group compared to O controls and the O PCOS group compared to NO PCOS (O PCOS: 266.62 ± 121.5; O control: 199.78 ± 62.81; NO PCOS: 181.86 ± 48.7; p>0.05). These results support the association of ANGPTL3 with dyslipidemia and PCOS, especially in obesity.

Güneş et al.^(13)^ found that ANGPTL4 was elevated in women with PCOS and correlated positively with insulin resistance. Jiang et al.^(38)^ however, found no difference in ANGPTL4 expression between NW and O groups within both PCOS and control groups. Similarly, in our study, ANGPTL4 levels did not differ between NW, SO, and O PCOS and control groups. However, the O PCOS group had significantly higher ANGPTL4 levels compared to the NO PCOS group (p<0.05).

Previous studies have shown lower plasma omentin-1 levels in women with PCOS than in controls^(13,39,40)^ with negative correlations between omentin-1 and BMI, insulin, and androgen levels.^(13,39,41)^ In our study, omentin-1 was lower in the overall PCOS group and in the SO PCOS group compared to controls (p<0.05), consistent with these findings. We also observed a lower omentin-1 level in the NW PCOS group compared to NW controls, although not statistically significant. Additionally, omentin-1 showed significant positive correlations with CRP, TNF-α, IL-6, HbA1C, LDL, total cholesterol, WHR, and body composition measures, contrasting with other studies that reported a negative correlation between omentin-1 and IL-6 and TNF-α in PCOS.^(21)^

Jalilian et al.^(18)^ found significantly higher serum leptin levels in women with PCOS compared to controls, with no association with insulin levels. Seth et al.'s^(42)^ meta-analysis suggested that while leptin levels are generally higher in PCOS, the relationship remains controversial. A recent study also found elevated serum leptin levels in PCOS, particularly in hyperandrogenemic and overweight/obese subgroups, associating higher leptin levels with PCOS.^(43)^ In our study, leptin was significantly elevated in the O PCOS group compared to controls, supporting its role in PCOS prediction. We found significant positive correlations between leptin and BMI, HbA1C, LDL, and total cholesterol in PCOS patients.

The conflicting data in the literature may stem from variations in study designs, analysis methods, sample sizes, and ethnic backgrounds. Additionally, proteolytic fragments, in addition to full-length proteins, might have functional roles that influence analysis results.^(44–48)^ Most studies have either focused on overweight/obese or lean PCOS patients and have not conducted subgroup analyses, though BMI can substantially affect circulating protein levels. Wu et al.^(33)^ also found that PCOS patients carrying the CT+TT genotype had higher ANGPTL8 levels than those with the CC genotype. Therefore, polygenic inheritance and gene polymorphisms in PCOS can further complicate the interpretation of protein study results.

Our study has several limitations. First, the patient sample size was relatively small, which could lead to statistical limitations. Second, we did not account for the nutritional status or physical activity levels of the participants. Third, the study was conducted within a single ethnic group. However, our study also offers some advantages: unlike previous studies, we evaluated not only lean and obese PCOS patients but also three subgroups NW, SO, and O. Additionally, we explored the predictive power of a combination of variables for PCOS by constructing a regression model using ANGPTL, cytokines, and inflammation markers, while addressing multicollinearity issues.

## Conclusion

Our study identified a model combining ANGPTL3, 4, 8, inflammatory cytokines (IL-6, TNF-α), and omentin-1 as predictive of PCOS. The model revealed that lower levels of omentin-1 and higher levels of leptin and CRP are associated with a higher risk of PCOS. These findings suggest that ANGPTLs play a role in PCOS, possibly by interacting with chronic low-grade inflammation. Future studies with larger sample sizes and ethnically diverse populations are needed to validate these results and investigate the molecular mechanisms by which ANGPTLs contribute to the pathophysiology of PCOS.
